# Effects of de-implementation strategies aimed at reducing low-value nursing procedures: a systematic review and meta-analysis

**DOI:** 10.1186/s13012-020-00995-z

**Published:** 2020-05-25

**Authors:** Tessa Rietbergen, Denise Spoon, Anja H. Brunsveld-Reinders, Jan W. Schoones, Anita Huis, Maud Heinen, Anke Persoon, Monique van Dijk, Hester Vermeulen, Erwin Ista, Leti van Bodegom-Vos

**Affiliations:** 1 Department of Biomedical Data Sciences, Section Medical Decision Making, Leiden University Medical Center, Leiden, The Netherlands; 2 Department of Internal Medicine, Nursing Science, Erasmus MC University Medical Center, Rotterdam, The Netherlands; 3 Department of Quality and Patient Safety, Leiden University Medical Center, Leiden, The Netherlands; 4 Leiden University Medical Center, Walaeus Library, Leiden, The Netherlands; 5 Department of Primary and Community Care, Radboud Institute for Health Sciences, Scientific Institute for Quality of Healthcare, Radboud University Medical Center, Nijmegen, The Netherlands; 6 Faculty of Health and Social Studies, HAN University of Applied Sciences, Nijmegen, The Netherlands

**Keywords:** Nursing, Low-value care, De-implementation, Deprescription, Health services Misuse, Inappropriate prescribing

## Abstract

**Background:**

In the last decade, there is an increasing focus on detecting and compiling lists of low-value nursing procedures. However, less is known about effective de-implementation strategies for these procedures. Therefore, the aim of this systematic review was to summarize the evidence of effective strategies to de-implement low-value nursing procedures.

**Methods:**

PubMed, Embase, Emcare, CINAHL, PsycINFO, Cochrane Central Register of Controlled Trials, Web of Science, and Google Scholar were searched till January 2020. Additionally, reference lists and citations of the included studies were searched. Studies were included that described de-implementation of low-value nursing procedures, i.e., procedures, test, or drug orders by nurses or nurse practitioners. PRISMA guideline was followed, and the ‘Cochrane Effective Practice and Organisation of Care’ (EPOC) taxonomy was used to categorize de-implementation strategies. A meta-analysis was performed for the volume of low-value nursing procedures in controlled studies, and Mantel–Haenszel risk ratios (95% CI) were calculated using a random effects model.

**Results:**

Twenty-seven studies were included in this review. Studies used a (cluster) randomized design (*n* = 10), controlled before-after design (*n* = 5), and an uncontrolled before-after design (*n* = 12). Low-value nursing procedures performed by nurses and/or nurse specialists that were found in this study were restraint use (*n* = 20), inappropriate antibiotic prescribing (*n* = 3), indwelling or unnecessary urinary catheters use (*n* = 2), ordering unnecessary liver function tests (*n* = 1), and unnecessary antipsychotic prescribing (*n* = 1). Fourteen studies showed a significant reduction in low-value nursing procedures. Thirteen of these 14 studies included an educational component within their de-implementation strategy. Twelve controlled studies were included in the meta-analysis. Subgroup analyses for study design showed no statistically significant subgroup effect for the volume of low-value nursing procedures (*p* = 0.20).

**Conclusions:**

The majority of the studies with a positive significant effect used a de-implementation strategy with an educational component. Unfortunately, no conclusions can be drawn about which strategy is most effective for reducing low-value nursing care due to a high level of heterogeneity and a lack of studies. We recommend that future studies better report the effects of de-implementation strategies and perform a process evaluation to determine to which extent the strategy has been used.

**Trial registration:**

The review is registered in Prospero (CRD42018105100).

Contribution to literature

- Educational strategies are most frequently used to de-implement low-value nursing procedures in daily practice.
- The level of evidence for de-implementation strategies in nursing is limited due to a lack of high-quality studies.
- More high-quality research is needed to asses which de-implementation strategies are the most effective for reducing low-value nursing procedures.

## Background

Health care professionals intentionally or unintentionally order tests, treatments, and perform procedures on a daily basis that offer little or no benefit to patient care. This low-value care is proven to be ineffective or has not been proven to be effective, can even harm patients, and waste valuable resources [1–3]. In addition, it wastes time that the health care professional can spend on more effective practices or care that is left undone [4, 5]. The Institute of Medicine estimates that up to 30% of care provided in the USA is wasted on low-value care [6]. If even a fraction of this low-value care could be eliminated, the resulting quality improvement and cost savings would be transformational [7].

Most initiatives to eliminate low-value care are mainly focused on care provided by doctors [8], but many low-value procedures are also routinely performed by nurses [4, 9]. Well-known examples of low-value nursing procedures include the use of physical restraints in patients with a delirium, the use of bandages for wounds closed by primary intention, and performing a bladder washout [4]. Since nurses are the largest group of health care providers [4], there is a great potential in improving quality of care by involving and targeting them in de-implementation initiatives [4, 10]. As a first step to reduce low-value nursing procedures, ‘Choosing Wisely’ lists of nursing procedures are recently created in several countries [1, 4, 11, 12]. The next step is to translate these ‘Choosing Wisely’ lists into action [13]. To actually reduce the use of low-value nursing procedures, awareness should be created for the ‘Choosing Wisely’ lists and effective de-implementation strategies need to be developed and executed [7, 14, 15]. These de-implementation strategies should be theory- and evidence-based and informed by analysis of barriers and facilitators that influence the use of low-value care, since this is expected to increase the adherence, adoption, and effectiveness of these de-implementation strategies [5, 16, 17].

A previous systematic review performed by Colla et al. [7] already reveals that multifaceted de-implementation strategies targeted at health care providers and patients have the greatest potential to reduce the use of low-value care. Besides, clinical decision support tools, performance feedback and education (alone or as part of a multifaceted strategy), are promising strategies for reducing low-value care. However, Colla et al. [7] also noted that little is known about interventions directed at non-physician staff members such as nurses, and that most interventions targeted at non-physician staff are aimed at assisting physician’s decision-making. So, it is still unknown whether the conclusions about effective de-implementation strategies also apply for the reduction of low-value nursing procedures. Since nurses might have other learning styles than physicians [18], other strategies could be more effective to de-implement low-value nursing procedures. Therefore, the aim of this systematic review is to summarize the evidence of effective de-implementation strategies aiming to reduce or eliminate low-value nursing procedures.

## Methods

This systematic review was conducted according to the Preferred Reporting Items for Systematic Reviews and Meta-analyses (PRISMA) [19]. The review protocol was registered in the PROSPERO database of systematic reviews (registration number: CRD42018105100, (www.crd.york.ac.uk/prospero/display_record.php?RecordID=105100).

### Search strategy

To identify all eligible studies reporting on effective de-implementation strategies aiming to reduce low-value nursing procedures, a systematic literature search was performed in PubMed, Embase, Emcare, CINAHL, PsycINFO, Cochrane Central Register of Controlled Trials, Web of Science, and Google Scholar. The full search strategy is included in Additional file 1. The search was limited to the literature published till January 2020. Search terms were based on 43 unique terms for de-implementation that were used for the process of reducing low value care found by Niven et al. [20], and there were no language or other search filter limits. After the initial search, the reference lists and citations of all included studies were explored to find more relevant studies. An expert health librarian at the Leiden University Medical Center guided the search.

### Selection of studies

Two researchers (TR, AB, or LvB) first independently reviewed title and abstract of the studies, followed by full texts review. If there was no consensus between the two reviewers and differences could not be solved by discussion, a third reviewer was consulted.

Studies were eligible for inclusion in the systematic review if they fulfilled the following inclusion criteria:

- Focus of the study: reduction of low-value nursing procedures. Low-value nursing procedures were in this review defined as actual treatments and actions that are unlikely to benefit the patient given the harms, costs, available alternatives, or preferences of patients, and are initiated independently by a nurse and/or nurse specialist (i.e., without an order of another health care provider).
- Type of study: all studies that use a reference group (including pre-post comparisons), i.e., randomized controlled trials, cluster randomized trials, quasi-randomized controlled trials, non-randomized controlled trials, controlled before-after studies, interrupted time series studies, or uncontrolled before-after studies.
- Setting: hospitals, nursing homes, long-term care facilities, and community settings.
- Outcome: the study had to report on the effect of the de-implementation strategy on the volume of low-value nursing procedures.

Case studies of individual patients, letters, and editorials were excluded. Controlled studies were included in the meta-analysis if they reported data on the change in volume of low-value nursing procedures or if this data was available to the researchers after sending a request to the authors of the included paper.

### Data extraction

Data of the included studies was extracted in a standardized data extraction form in Microsoft Access (version 2016) by one researcher (TR or AB). A second researcher (TR, AB, or DS) independently checked the extracted data. Any discrepancy was resolved by discussion between the researchers until consensus was reached. If this was not possible, a third researcher (LvB) made a judgment on the data entered. The following information was collected from all included studies: country of origin, design, setting, location of care, type of low-value nursing procedure, de-implementation strategy based on barrier assessment, de-implementation strategies, participants, reimbursement and funding, primary and secondary outcomes. The primary outcome was the change in volume of the low-value nursing procedure. The secondary outcomes were adherence to the de-implementation strategy, changes in patient outcomes (e.g., pain), changes in patient satisfaction with care, changes in costs due to de-implementation of low-value nursing procedures, and changes in costs of the delivery of care. Authors of the included studies were contacted when more information was needed about unreported or missing data, and about the bias issues. If they did not respond, we sent a reminder after 2 to 6 weeks. We used the ‘Cochrane Effective Practice and Organisation of Care’ (EPOC) taxonomy [21] to categorize the different types of de-implementation strategies. The EPOC taxonomy includes four categories of strategies: (a) delivery arrangements, (b) financial arrangements, (c) governance arrangements, and (d) implementation strategies.

The quality of the studies was assessed by using two risk of bias tools by two independent researchers (TR, AB, or DS). The Cochrane Effective Practice and Organisation of Care (EPOC) [22] was used for studies with a separate control group (randomized trails, and controlled before-after studies), and the Newcastle-Ottawa Scale (NOS) [23] was used for uncontrolled studies. The EPOC tool consists of nine suggested risk of bias criteria: random sequence generation, allocation concealment, baseline outcome measurements similar, baseline characteristics similar, incomplete outcome data, knowledge of the allocated interventions adequately prevented during the study, protection against contamination, selective outcome reporting, and other risks of bias. Every criterion was scored with low, high, or unclear risk. The NOS consists of three categories: (a) selection, (b) comparability, and (c) outcome. A certain number of stars could be given for each category, resulting in a score of good, fair, or poor quality of the studies. Disagreements in the risk of bias scoring were resolved by consensus or by discussion with a third researcher (TR, LvB, or DS).

### Statistical analyses

To summarize the overall evidence of de-implementation strategies aiming to reduce low-value nursing procedures in a descriptive and narrative synthesis, the data from all included studies was extracted in Microsoft Access (version 2016) and analyzed in Microsoft Excel (version 2016). The synthesis is performed separately for controlled and uncontrolled studies to reduce the risk of selection bias. To assess the effectiveness of de-implementation strategies to reduce low-value nursing procedures, data of the controlled studies on the use of low-value care was analyzed in Review Manager 5.3. Data about the use of low-value nursing procedures was pooled using a random effects model of Mantel-Haenszel [24], and risk ratios were calculated with 95% confidence intervals. The *I*^2^ statistics of Higgins [25] was used to measure heterogeneity between the included studies, which can be interpreted as the percentage of the total variability in a set of effect sizes between trials in a meta-analysis. When the *I*^2^ was 50% or higher, we considered the results as a moderate or high level of heterogeneity [25]. If heterogeneity was present, subgroup analyses were performed. Subgroup analyses were performed by design of the study (RCT, Cluster RCT, and controlled studies), type of low-value care, and type of de-implementation strategy (single versus multifaceted, and type of strategy). Subgroup analyses by type of design were performed because failure to use adequately concealed random allocation can distort the apparent effects of care in either direction [26]. Subgroup analyses for type of low-value nursing procedure were performed because the characteristics of the type of low-value nursing procedure that needs to be de-implemented (including underlying evidence, advantages of practice, credibility, attractiveness, feasibility) could be of influence on the effectiveness of the de-implementation strategy. Subgroup analyses for type of de-implementation strategy (including single versus multifaceted strategies and type of strategy according to EPOC taxonomy) were performed since we wanted to learn which strategy is most effective. A subgroup for type of design, low-value nursing procedure, or de-implementation strategy was only performed when at least two studies with respectively the same design, low-value nursing procedure, or de-implementation strategy could be included in each subgroup. Finally, sensitivity analyses for the subgroups were performed without studies with a high-risk score on 3 or more risk of bias criteria of the EPOC tool. Funnel plots were created to assess the publication bias.

## Results

### Study selection

**Fig. 1:**
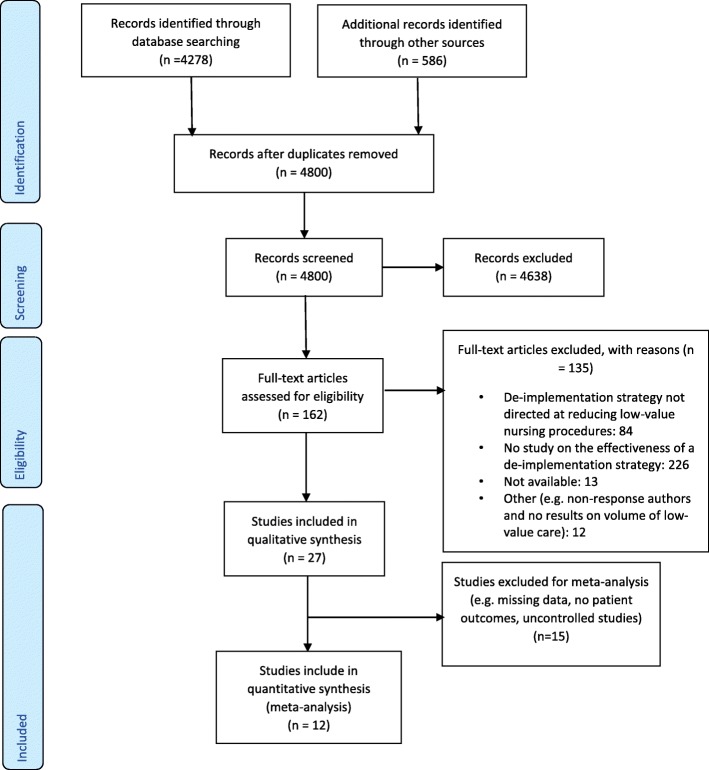
PRISMA flow diagram

The search strategy resulted in 4278 studies. The reference and citation search resulted in an additional 586 studies. After removing 64 duplicates, 4800 abstracts remained. After screening on title and abstract, 162 full texts were reviewed. A total of 27 studies were found to be eligible for inclusion (Fig. 1), including 12 uncontrolled studies [9, 27–37] and 15 controlled studies [38–52]. Reasons for exclusion were (1) de-implementation strategy was not directed at reducing low-value nursing procedures (but at low-value care provided by other health care professionals or at low-value nursing procedures that require an order of other health care professionals (*n* = 84) such as medication prescribing or requests for lab testing by physicians), (2) study does not include an assessment of the effectiveness of a de-implementation strategy (*n* = 26), (3) full text was not available (*n* = 13), and (4) other (e.g., non-response authors and no results reported on volume of low-value care) (*n* = 12).

### Quality of the included studies

The risk of bias of the uncontrolled studies (*n* = 12), estimated with the Newcastle-Ottawa scale, is shown in Table 1. Overall, the quality of the included uncontrolled studies was poor, mainly due to a low score on the comparability domain due to lack of matching of exposed and non-exposed individuals in the study design and/or a lack of correction for confounders in the analyses.

**Table 1:** Risk of Bias Newcastle-Ottawa Scale (NOS) of uncontrolled studies (*n*=12)

Author	Score selection	Score comparability	Score outcome	Conclusion
Alexaitis et al. 2014 [27]	★★★★	-	-	Poor
Amato et al. 2006 [28]	-	-	★	Poor
Andersen et al. 2017 [29]	★★	-	★	Poor
Davis et al. 2008 [30]	★★★	-	★	Poor
Eskandaria et al. 2018 [31]	★★	★	-	Poor
Hevener et al. 2016 [32]	-	-	-	Poor
Link et al. 2016 [33]	★★★★	-	-	Poor
McCue et al. 2004 [34]	★	-	★★	Poor
Mitchell et al. 2018 [9]	-	-	★★	Poor
Sinitsky et al. 2017 [35]	★	-	★	Poor
Thakker et al. 2018 [36]	★★★	-	★	Poor
Weddle et al. 2016 [37]	★★	-	★★★	Poor

Poor quality; 0 or 1 star in selection domain OR 0 stars in comparability domain OR 0 or 1 stars in outcome/exposure domain. Fair quality: 2 stars in selection domain AND 1 or 2 stars in comparability domain AND 2 or 3 stars in outcome/exposure domain. Good quality: 3 or 4 stars in selection domain AND 1 or 2 stars in comparability domain AND 2 or 3 stars in outcome/exposure domain [23]

**Fig. 2:**
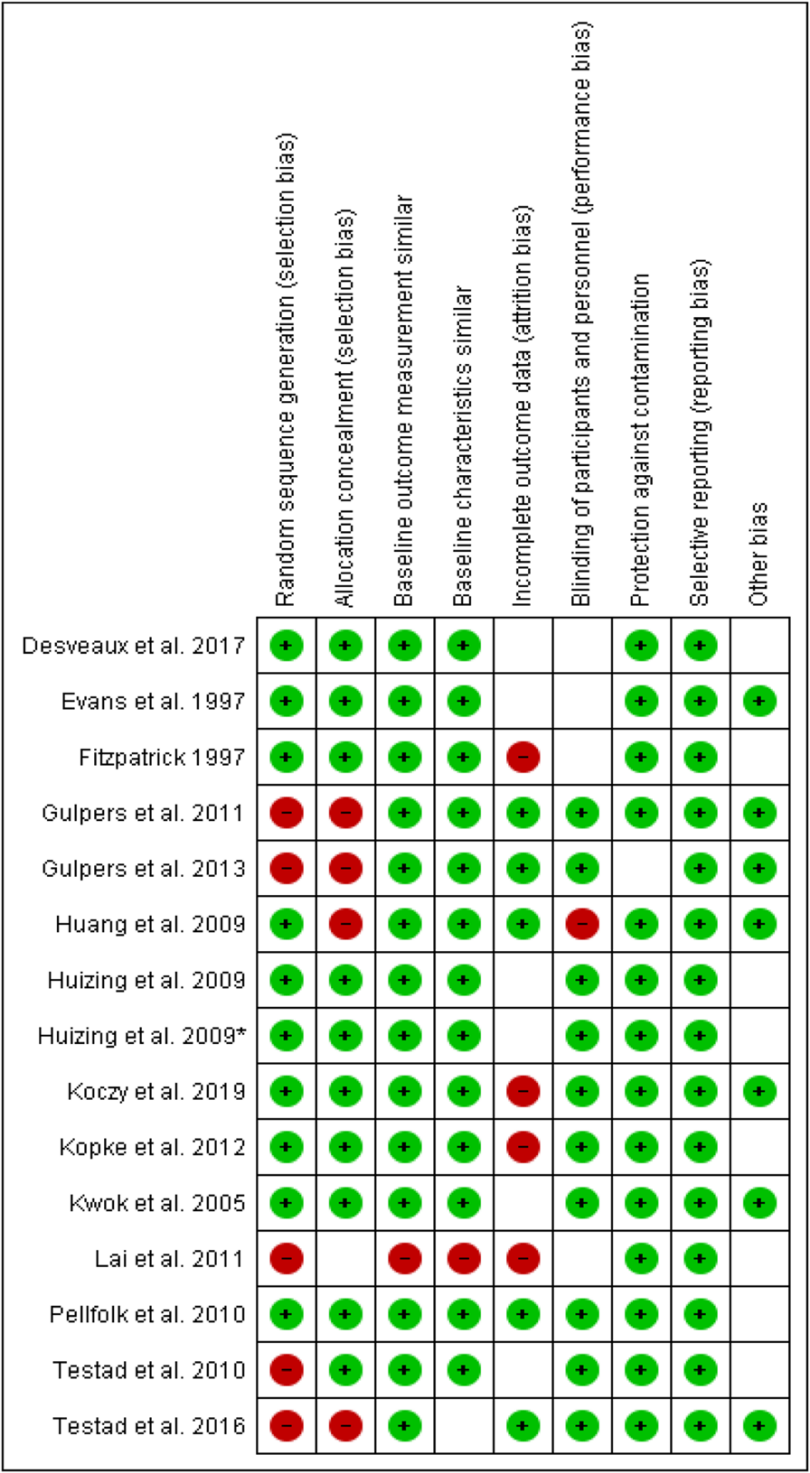
Risk of bias Cochrane Effective Practice and Organisation of Care (EPOC) of controlled studies (*n* = 15). Randomization: low risk if randomization method is described. Allocation concealment: low risk if unit of allocation was by team/institution OR by patient with some kind of randomization method. Baseline measurement similar: low risk if baseline measurements were performed and no important difference present across groups OR imbalanced but appropriate adjusted. Baseline characteristics similar: low risk if characteristics were reported and similar. Incomplete outcome data: low risk if missing outcomes were unlikely to bias the results. Blinding: low risk if the authors stated blind assessment OR objective outcomes. Contamination: low risk if allocation was by team/ institution/practice and unlikely control group received intervention. Selective reporting: low risk if there is no evidence that outcomes were selectively reported. Other: low risk if there is no evidence of other risk of bias. Green circle: low risk of bias, red circle: High risk of bias, empty box: unclear risk of bias

The risk of bias of the controlled studies (*n* = 15), scored with EPOC, showed that nine studies scored low risk on seven of the nine risk of bias criteria (Fig. 2). For only five studies [41–43, 50, 52], the missing outcomes were unlikely to bias the results. For the other studies, there was an unclear or high risk for missing outcomes that were likely to bias the results [38–40, 44–49, 51]. Three studies did not perform statistical tests for measuring the effect of their de-implementation strategy [9, 28, 36].

### Study characteristics

#### Uncontrolled studies

Twelve of the 27 studies (44%) [9, 27–37] had an uncontrolled before-after design (Table 2). Of these 12 studies, six focused their intervention on reducing restraint use [9, 28, 29, 31, 32, 34], three on reducing inappropriate antibiotic prescribing [30, 33, 37], two on reducing time of indwelling urinary catheters [27, 36], and one on reducing unnecessary liver function tests [35]. The de-implementation strategy used within the uncontrolled studies were directed at nursing staff working in a hospital (*n* = 10) [9, 27–32, 34–36] and in an urgent care center (*n* = 2) [33, 37]. Most of the uncontrolled studies had a single center design (*n* = 9) and were performed in North America (*n* = 9) [9, 27, 28, 30, 32–34, 36, 37]. Most uncontrolled studies did not report on the characteristics of the patients and/or on the characteristics of the health care providers. Four uncontrolled studies (33%) have not clearly described the duration of the intervention [37, 38, 48, 50]. For the uncontrolled studies that mentioned the duration of the intervention, it differed from 2 to 14 months. The follow up time after de-implementation of the studies that reported these results differed from 1 month follow up till 12 months.

**Table 2:** Design and characteristics of uncontrolled studies (*N* = 12)

Author (year), Country	Design study	Setting	Target group	Type of low value care	Primary outcome (s)	Before	After	Difference/ statistical test results	Statistical analyses performed (Yes/No)	Positive significant effect (*p* ≤ 0.05) (Yes/No)
Alexaitis et al. (2014), USA [27]	Uncontrolled Before-after	Hospital	ICU nurses	Catheter use	The average percentage of catheter utilization	74.14%	76.2%	2.08%	Yes	No
Amato et al. (2006), USA [28]	Uncontrolled Before-after	Hospital	Nurses	Restraint use	The percentage of restraint use (2 units)	–	–	− 29.2% stroke rehabilitation unit, -16,2% brain injury unit	No	/
Andersen et al. (2017), Denmark [29]	Uncontrolled Before-after	Hospital	Nurses	Restraint use	The percentage of restraint use	–	–	− 38%	Yes	No
Davis et al. (2008), USA [30]	Uncontrolled Before-after	Hospital	Nurse Practitioner	Antibiotic prescribing	The rate of antibiotic prescribing	–	–	*F* = 0.076	Yes	No
Eskandaria et al. (2018), Malaysia [31]	Uncontrolled before-after	Hospital	Nurses	Restraint use	The incidence rate of physical restraint use	5.57%	3.81%	− 1.76%	Yes	Yes
Hevenver et al. (2016), USA [32]	Uncontrolled-before after	Hospital	ICU nurses	Restraint use	The incidence rate of restraint use	–	–	− 32%	Yes	Yes
Link et al. (2016), USA [43]	Uncontrolled Before-after	Urgent care center	Nurse practitioner (NP) and Physician Assistant (PA)	Antibiotic prescribing	The rate of antibiotic prescribing	91.7%	29.8%	− 61.9%	Yes	Yes
McCue et al. (2004), USA [34]	Uncontrolled before-after	Hospital	Psychiatric nurses	Restraint use	The number of restraints/1000 patient-days	0.8%	0.4%	− 0.4%	Yes	Yes
Mitchell et al. (2018), USA [9]	Uncontrolled Before-after	Hospital	ICU nurses	Restraint use	The rate of restraint use	61%	31%	− 30%	No	/
Sinitsky et al. (2017), UK [35]	Uncontrolled Before-after	Hospital	Pediatric intensive care nurses	Liver function tests (LFT)	Total number of LFTs per bed day	N/A	N/A	N/A	Yes	Yes
Thakker et al. (2018), Canada [36]	Uncontrolled Before-after	Hospital	Orthopaedic nurses	Catheter use	The average rate of indwelling catheter use	55.2%	19.8%	− 35.4%	No	/
Weddle et al. (2016), USA [37]	Uncontrolled Before-after	Urgent care center	Nurse practitioner	Antibiotic prescribing	The rates of inappropriate antibiotic prescribing per month	9%	6%	− 3%	Yes	Yes

*N*/*A* not available

#### Controlled studies

Fifteen of the 27 studies (56%) had a controlled design, including three RCTs (11%), seven cluster RCTs (26%), and five controlled before-after designs (19%) (Table 3). Of the controlled studies, 14 studies focused their intervention on reducing restraint use [39–52], and one on reducing inappropriate antipsychotic prescribing [38]. The de-implementation strategy used within the controlled studies were directed at nursing staff working in a nursing home (*n* = 10) [38, 39, 41, 42, 44–47, 51, 52], in a hospital (*n* = 4) [40, 43, 48, 49], and in a residential care facility (*n* = 1) [50]. Most of the controlled studies had a multicenter design (*n* = 12) and were performed in Europe (*n* = 9). Not all controlled studies reported on the patients’ characteristics and/or on the characteristics of the health care providers. Three controlled studies (20%) have not clearly described the duration of the intervention [27, 31, 39]. For the controlled studies that mentioned the duration of the intervention, it differed from 1 to 12 months. The follow up time after de-implementation of the studies that reported these results differed from no follow up till 24 months.

**Table 3:** Design and characteristics of controlled studies (*N* = 15)

Author (year), Country	Design study	Setting	Target group	Type of low value care	Primary outcome (s)	Posttest intervention group (%)^a^	Posttest Control group (%)^a^	Statistical analyses performed (Yes/No)	Positive significant effect (Yes/No)
Desveaux et al. (2017) [38]^b^	Cluster RCT	Nursing home	Nurses	Antipsychotic prescribing (APM)	The days dispensed APM in the previous week	624/2947 (21.2%)	898/4162 (21.6%)	Yes	No
Evans et al. 1997, USA [39]	RCT	Nursing home	Gerontologic nurses	Restraint use	The prevalence of restraint use	18/127 (14.2%)	79/184 (42.9%)	Yes	Yes
Fitzpatrick (1997), USA [40]	Controlled before after	Hospital	Critical care and intermediate nurse	Restraint use	The incidence of restraint use	29/91 (31.9%)	8/51 (15.7%)	Yes	No
Gulpers et al. (2011), The Netherlands [41]	Controlled before after	Nursing home	Psychogeriatric nurses	Restraint use	The rate of residents with at least 1 physical restraint	135/250 (54.0%)	107/155 (69.0%)	Yes	Yes
Gulpers et al. (2013), the Netherlands [42]	Controlled before after	Nursing home	Psychogeriatric nurses	Restraint use	The rate of residents with at least 1 physical restraint	80/134 (59.7%)	68/91 (74.7%)	Yes	Yes
Huang et al. (2009) [43]^c^	Controlled before after	Hospital	Nurses	Restraint use	The reported Practice of Physical Restraint Use	40.88	39.20	Yes	Yes
Huizing et al. (2009), The Netherlands [44]	Cluster RCT	Nursing home	Nurses and registered Nurses	Restraint use	The use of restraints per residents	25/53 (47.2%)	15/37 (40.5%)	Yes	No
Huizing et al. (2009), The Netherlands [45]	Cluster RCT	Nursing home	Nurses	Restraint use	The use of restraints per residents	81/126 (64.3%)	69/115 (60.0%)	Yes	No
Koczy et al. (2011), Germany [46]	Cluster RCT	Nursing home	Nurses	Restraint use	The complete cessation of restraint use	173/208 (83.2%)	114/125 (91.2%)	Yes	No
Kopke et al. (2012), Germany [47]	RCT	Nursing home	Nurses	Restraint use	The percentage of residents with at least 1 physical restraint	423/1868 (22.6%)	525/1802 (29.1%)	Yes	Yes
Kwok et al. (2005), China [48]^b^	RCT	Hospital	Geriatric nurses	Restraint use	The proportion of subjects ever restrained	N/A	N/A	Yes	No
Lai et al. (2011), China [49]	Controlled before after	Hospital	Nurses	Restraint use	The prevalence of restraint use	299/612 (48.9%)	21/155 (13.5%)	Yes	No
Pellfolk et al. (2010), Sweden [50]	Cluster RCT	Residential care facilities	Registered nurses, licensed practical nurses and nurse’s aides	Restraint use	The use of restraint use	30/149 (20.1%)	53/139 (38.1%)	Yes	Yes
Testad et al. (2010), Norway [51]^b^	Cluster RCT	Nursing home	Nurses	Restraint use	The use of restraint use	N/A	N/A	Yes	Yes
Testad et al. (2016), Norway [52]	Cluster RCT	Nursing home	Nurses	Restraint use	The use of restraint use	15/83 (18.1%)	10/114 (8.8%)	Yes	Yes

*N*/*A* not available

^a^Numbers based on the extracted results used for the meta-analyses

^b^Data for meta-analyses not available

^c^Data was not measured at patient level

### Strategies to reduce low-value care

#### Uncontrolled studies

The de-implementation strategies of six uncontrolled studies resulted in a positive significant effect on the volume of low-value nursing procedures (Table 2). The reduction in volume of low-value nursing procedure in the uncontrolled studies with a positive significant effect and with available data (*n* = 5) ranged from 0.4% [34] reduction of low-value nursing care till 61.9% [33]. Four of the positive significant studies had a single de-implementation strategy [31, 33, 35, 37], which means that the strategies consisted of only one strategy component (Table 4). Five of the six studies used an educational component (meetings and/or materials) as an intervention strategy [31–34, 37]. However, none of the studies with a positive significant effect on the primary outcome based their de-implementation strategy on a barrier assessment. Only one uncontrolled study without a positive significant effect performed a barrier assessment [9].

**Table 4:** Type of intervention of the uncontrolled studies (*n* = 12)

Author (year)	Type of low-value care	Single or multifaceted intervention strategy	Interventions from the EPOC taxonomy													Description of intervention strategy (sorted by EPOC Taxonomy)	Positive Significanteffect (*p* ≤ 0.05) (Yes/No)
			E	AF	P	C	CQ	H	L	M	MP	O	S	T	TI		
Alexaitis et al. 2014 [27]	Catheter use	Multifaceted	X	X		X			X							Educational meetings: - Education about alternatives to indwelling catheters and routine catheter care - Education about the protocol - Didactic education encompassed routine catheter maintenance, bedside bladder ultrasound indications, and criteria in the nurse-driven protocol. Simulation education to assess proficiency in using the bladder ultrasonography was provided to nurses by the clinical leaders and charge nurses Audit and Feedback: - Compliance monitoring to ensure adherence to the protocol and guidelines for routine catheter care - Analysis of identified CAUTIs - Daily catheter rounds to assess the need for catheter continuation Clinical guidelines: - Evidence-based, nurse-driven protocol for urinary catheter management Local consensus processes - Protocol approval by NSICU stakeholders	No
Amato et al. 2006 [28]^a^	Restraint use	Multifaceted	X	X					X							Educational meetings: - Formal and informal information sessions for all levels of nursing staff about the restraint and seclusion policy as well as the hospital’s philosophy regarding restraint use - A local vendor demonstrated restraint alternatives - Training on proper use of the devices Educational outreach visits: - Consultation rounds of a clinical nurse specialist Audit and feedback: - The nurses’ adherence to the plan of care was monitored and reviewed during the ongoing consultation rounds, at which time individual nurse-to-nurse feedback was provided - The quality management department provided aggregate data in the form of monthly run charts for fall rates and physical restraint use on each unit Local consensus processes: - The administrative component involved gaining the active support of the director of nursing, nurse managers, patient care coordinators, physician leaders, and therapists prior to implementation of the program	/
Andersen et al. 2017 [29]	Restraint use	Multifaceted	X										X			Educational meetings: - Education by occupational therapists. The occupational therapists on the project unit completed a 3-day course and a 1-day workshop with the rest of the staff four months later Sensory modalities for the patient: - Access to a variety of sensory modalities located in the unit and a sensory room	No
Davis et al. 2008 [30]	Antibiotic prescribing	Multifaceted	X	X												Educational meetings: - The standards of care for the treatment of a viral upper respiratory tract infections were presented to the individual health care provider Audit and feedback: - Thirty randomly selected charts coded by the individual healthcare providers - Individual provider and group statistics regarding rates of prescribing.	No
Eskandaria et al. 2018 [31]	Restraint use	Single	X													Educational meetings: - Lectures - Group discussion - Demonstration on some types of physical restraint and proper use of physical restraint - Three video demonstrations	Yes
Hevenver et al. 2016 [32]	Restraint use	Multifaceted	X					X								Educational meetings: - 1-on-1 discussion about proper use of restraints and alternatives Educational materials: - Online educational activity Health information system: - Restraint decision tool	Yes
Link et al. 2016 [33]	Antibiotic prescribing	Single	X													Educational meetings: - The intervention consisted of a 60-min face to-face interactive provider education activity. - Small group discussion - Case studies with didactic lecture - Treatment algorithms	Yes
McCue et al. 2004 [34]	Restraint use	Multifaceted	X				X		X					X		Educational materials: - All clinical staff on the psychiatric inpatient service received training on crisis intervention techniques that can be used as an alternative to restraint (videotapes) - A stress/anger management group for patients was added to the inpatient service's therapeutic programming. Continuous quality improvement: - Daily review of all restraints Local Consensus processes: - Identification of restraint prone patients Team: - Crisis response team - Incentive system for the staff	Yes
Mitchell et al. 2018 [9]^a^	Restraint use	Multifaceted	X								X					Educational meetings: - Presentations Educational materials: - Flyers - Posters Monitoring the performance of the delivery of healthcare: - Monthly prevalence is determined on all units by bedside nurses. If a patient has restraints in place, the patient’s chart is reviewed for orders and proper documentation	/
Sinitsky et al. 2017 [35]	Liver function tests	Single						X								Health Information System: - Blood test form	Yes
Thakker et al. 2018 [36]^a^	Catheter use	Multifaceted	X	X												Educational meetings: - Education about the guidelines to ensure adherence and to standardize the criteria for catheter use. Audit and Feedback: - Reminders about adhering to the CAUTI prevention guidelines in daily safety huddles and weekly staff meetings	/
Weddle et al. 2016 [37]	Antibiotic prescribing	Single	X													Educational meetings: - Educational session used evidence-based guidelines and a local antibiogram to provide specific recommendations for the best prescribing practices	Yes

Intervention strategies are classified using the EPOC Taxonomy [21]: *E* education (meetings, materials, games, and outreach visits), *AF* audit and feedback, *P* packages of care, *C* clinical guidelines, *CQ* continuous quality improvement, *H* health information system, *L* local consensus processes, *M* monitoring, *MP* monitoring the performance of the delivery of healthcare, *O* organizational culture, *S* sensory modalities for patients, *T* team, *TI* tailored interventions

^a^No statistical testing

None of the uncontrolled studies reported about adherence to the de-implementation strategy, changes in patient satisfaction with care, changes in costs made by the de-implementation strategy, and changes in costs of the delivery of care.

#### Controlled studies

The de-implementation strategies of eight of the 15 controlled studies resulted in a positive significant effect on volume of low-value nursing procedures (Table 3). The reduction in volume of low-value nursing procedure in the controlled studies with a positive significant effect who measured patient outcomes (*n* = 7) ranged from 6.5% [47] till 28.7% [39]. Seven of the eight positive significant studies had a multifaceted de-implementation strategy (Table 5) and all eight studies focused their strategy at reducing the use of restraints [39, 41–43, 47, 50–52]. Besides, the eight studies with a positive significant effect had an educational component (educational meetings, educational materials, educational outreach visits, and educational games) in their de-implementation strategy. However, none of the studies with a positive significant effect on the primary outcome based their de-implementation strategy on a barrier assessment. Only one controlled study without a positive significant effect performed a barrier assessment [38].

**Table 5:** Type of intervention of the controlled studies (*n* = 15)

Author (year)	Type of low-value care	Single or multifaceted intervention strategy	Interventions from the EPOC taxonomy													Description of intervention strategy (sorted by EPOC Taxonomy)	Positive significant effect (*p* ≤ 0.05) (Yes/No)
			E	AF	P	C	CQ	H	L	M	MP	O	S	T	TI		
Desveaux et al. 2017 [38]	Antipsychotic prescribing	Multifaceted	X													Educational outreach visits: - Academic detailing (educational outreach) intervention delivered by registered health professionals following an intensive training program including relevant clinical issues and techniques to support health professional behavior change Educational materials: - Online practice reports	No
Evans et al. 1997 [39]	Restraint use	Single and multifaceted	X													Restraint education (RE) group Educational meetings: - Intensive education by a masters-prepared gerontologic nurse on restraint use Restraint education-with-consultation (REC) group Educational meetings: - Intensive education by a masters-prepared gerontologic nurse Educational outreach visits: - Unit-based nursing consultation	Yes
Fitzpatrick 1997 [40]	Restraint use	Single and Multifaceted (2 groups)	X													Single faceted group Educational materials: - Educational program: restraint education in service administered in the form of a self-learning module and the option to construct a poster in each unit Multifaceted group Educational materials: - Educational program: restraint education in service administered in the form of a self-learning module and the option to construct a poster in each unit. - Critical care restraint decision guide (CCRDG).	No
Gulpers et al. 2011 [41]	Restraint use	Multifaceted	X						X							Educational meetings: - Nursing home staff education - Availability of alternative interventions Educational outreach visits: - Consultation by a nurse specialist aimed at nursing home staff Local consensus processes: - Promotion of institutional policy change that discourages use of belt restraint	Yes
Gulpers et al. 2013 [42]	Restraint use	Multifaceted	X						X							Educational meetings: - Intensive educational program offered by two registered nurses with extensive experience in physical restraint reduction - Availability of alternative interventions Educational outreach visits:- Consultation from the two nurse specialists (who delivered the educational program) to individual nurses on the intervention wards Local consensus processes: - Policy change by the nursing home management, with new use of belts prohibited and current use reduced	Yes
Huang et al. 2009 [43]	Restraint use	Single	X													Educational meetings: - Power-Point presentations - Discussion - Scenario reflections	Yes
Huizing et al. 2009 [45]	Restraint use	Multifaceted	X													Educational meetings: - Educational program Educational outreach visits: - Consultation with a nurse specialist	No
Huizing et al. 2009 [44]	Restraint use	Multifaceted	X													Educational meetings: - Educational program Educational outreach visits: - Consultation with a nurse specialist	No
Koczy et al. 2011 [46]	Restraint use	Multifaceted	X					X							X	Educational meetings: - The training course included information on epidemiology, the side effects of restraint use, legal aspects and alternatives Health information system: - Technical aids, such as hip protectors and sensor mats Tailored interventions: - Problem-Solving Tools - Advice by telephone from the research team	No
Kopke et al. 2012 [47]	Restraint use	Multifaceted	X													Educational meetings: - Group sessions for all nursing staff - Additional training for nominated key nurses Educational materials: - Supportive material for nurses, residents, relatives, and legal guardians.	Yes
Kwok et al. 2005 [48]	Restraint use	Multifaceted	X					X								Educational meetings: - Education about how to use of the bed-chair pressure sensors and the importance of restraint reduction in improving patients’ outcomes Health information system: - Bed-chair pressure sensors	No
Lai et al. 2011 [49]	Restraint use	Multifaceted	X									X				Educational meetings: - Staff education package Educational outreach visits: - Consult with the project team for uncertainties and on an individual Organizational Culture - The setup of a restraint reduction committee (RRC)	No
Pellfolk et al. 2010 [50]	Restraint use	Multifaceted	X													Educational meetings: - One volunteer from each unit attended the whole education program - Educational seminar Educational materials: - Videotaped lectures. Three of the lectures also included a clinical vignette presented in writing, which could be used for group discussions.	Yes
Testad et al. 2010 [51]	Restraint use	Multifaceted	X													Educational meetings: - Two day seminar - Monthly group guidance for six months Educational materials: - Teaching manual	Yes
Testad et al. 2016 [52]	Restraint use	Multifaceted	X													Educational meetings: - Two day seminar - Monthly seven step guidance groups for six months Educational materials: - Manual of the updated intervention and the seven-step guidance group - Poster DMP model	Yes

Intervention strategies are classified using the EPOC Taxonomy [21]: *E* education (meetings, materials, games, and outreach visits), *AF* audit and feedback, *P* packages of care, *C* clinical guidelines, *CQ* continuous quality improvement, *H* health information system, *L* local consensus processes, *M* monitoring, *MP* monitoring the performance of the delivery of healthcare, *O* organizational culture, *S* sensory modalities for patients, *T* team, *TI* tailored interventions

No statistical testing

None of the studies reported about adherence to the de-implementation strategy, changes in patient satisfaction with care, changes in costs made by the de-implementation strategy, and changes in costs of the delivery of care. Five studies aiming to reduce restraint use, reported about falls [39, 46–49]. However, different outcome measurements (e.g., risk of falls, total number of falls, fall related injuries, the proportion of those who suffered from one or more falls, and the percentages of falls) have been used for these studies.

### Effectiveness of de-implementation strategies (meta-analysis) of controlled studies

**Fig. 3:**
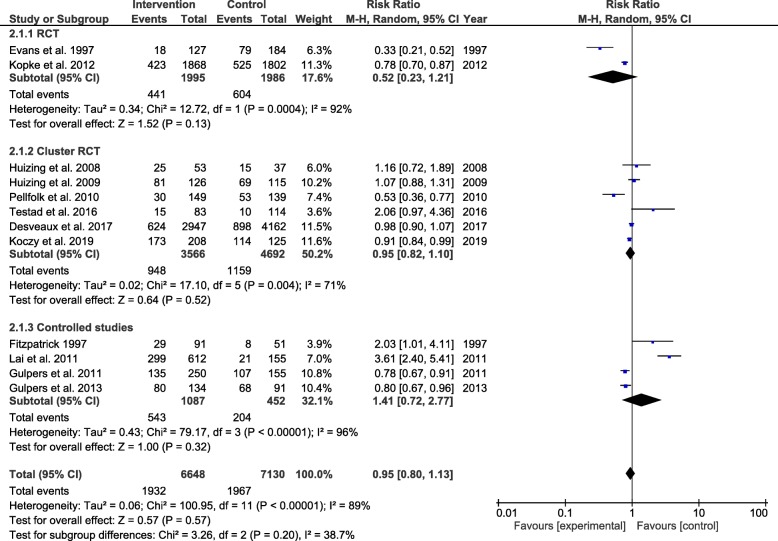
Subgroup analyses controlled studies: design study. *All studies included in the meta-analysis targeted their intervention at restraint use

The effectiveness of de-implementation strategies to reduce low-value nursing procedures is only assessed for the controlled studies. Twelve of the 15 controlled studies were eligible for inclusion in the meta-analyses [38–42, 44–47, 49, 50, 52]. Two controlled studies were excluded after no response of the author after sending a request for missing data [48, 51], and one study was excluded because the volume of low-value nursing procedures was not measured at patient level [43]. The relative risk ratio for the use of low-value nursing procedures for all 12 studies was 0.95 [95% CI 0.80, 1.13]. Considerable heterogeneity was present in the effect estimate (*I*^2^ = 89%) (Fig. 3). Subgroup analyses could only be performed for type of design (Fig. 3). A subgroup analysis for type of de-implementation strategy could not be performed due to a lack of studies with the same strategy. Also, a subgroup analyses for single vs. multifaceted strategies could not be performed due to a lack of studies with a single component strategy. A subgroup analyses for type of low-value care could not be performed due to a lack of studies assessing de-implementation strategies to reduce types of low-value nursing procedures other than restraint use.

**Fig. 4:**
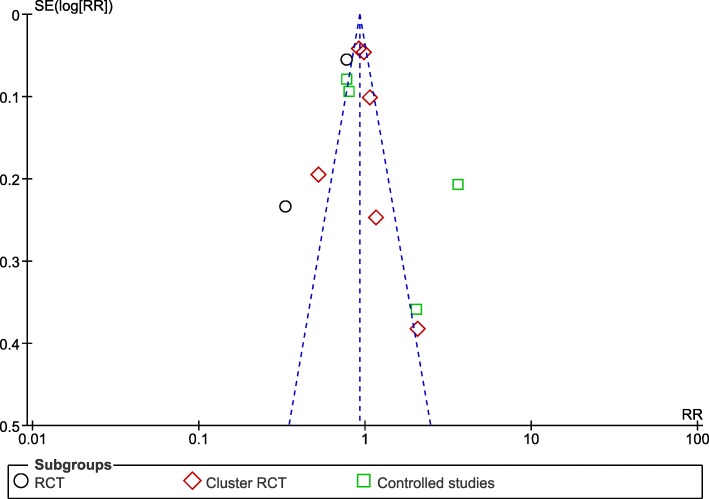
Funnel plot: design study

Subgroup analyses for the type of design of the studies (RCT, Cluster RCT, and controlled studies) showed no statistically significant subgroup effect (χ^2^ = 3.26, *p* = 0.20), a moderate level of heterogeneity between the studies (*I*^2^ = 39%), and a high level of heterogeneity within the subgroups (RCT = 92%, Cluster RCT = 71%, controlled studies = 96%) (Fig. 3). Based on the funnel plots, we suggest that there is no publication bias (Fig. 4).

## Discussion

To our knowledge, this is the first systematic review on de-implementation strategies for low-value nursing procedures. This systematic review identified both uncontrolled and controlled studies for the reduction of a limited range of low-value nursing procedures, namely physical restraint use, antibiotic and antipsychotic prescribing, requests for liver function tests, and urinary catheter use. The majority of the controlled and uncontrolled studies with a positive significant effect used a de-implementation strategy with an educational component (educational meetings, educational materials, educational outreach visits, and educational games) and focused their de-implementation strategy at reducing the use of restraints. An important difference between the controlled and uncontrolled studies with a positive significant effect is that the majority of the controlled studied used a multifaceted de-implementation strategy, and the majority of the positive significant uncontrolled studies used a single faceted de-implementation strategy. However, the use of educational components cannot be directly linked to successful de-implementation since both studies with a positive significant effect and studies without an effect or with a negative effect included these components. Due to heterogeneity and a lack of same strategies in the controlled studies, no conclusions can be drawn from the meta-analyses about the effectiveness of de-implementation strategies for low-value nursing procedures.

Despite increasing attention for the de-implementation of low-value nursing procedures, we only found 27 articles that we could include in our systematic review. However, the number of studies increased within the last decade. Only one study was found in the nineties, where seven studies were found from 2000 till 2010, and 18 studies from 2010 till 2020. This shows the attention for this important topic; however, more variation in the strategies to be evaluated is needed to get a full picture of effective or non-effective de-implementation strategies for nurses. Additionally, this study showed from the high number of excluded studies in which dependent nursing procedures are de-implemented, i.e., nursing procedures that require an order of another health care professional, that nurses have an important role in the de-implementation of low-value care. Due to differences in responsibilities in different countries, some nursing procedures are in some countries independently and in other countries dependently performed, for example the use of urinary catheters. As a consequence, some studies on this kind of topics are included in this review (as nurses are allowed independently to decide) or excluded (as nurses need an order for the nursing procedure).

The results of this systematic review showed some similarities and differences with previous findings in the literature regarding effective types of de-implementation strategies. A similarity is that our review showed as in a previous study of Colla et al. [7] that most studies used multifaceted strategies including an educational component. A difference with the study of Colla et al. [7] is that our review did not identify successful multifaceted de-implementation strategies that included a clinical decision support tool and/or performance feedback in their strategy. This may be the result of different inclusion criteria and focus of the study. While Colla et al. [7] focused on successful de-implementation strategies in health services, we only included studies that assessed the effectiveness of strategies to de-implement low-value nursing procedures.

To increase the effectiveness of de-implementation strategies, it is recommended in the literature to use a strategy which is geared at barriers and facilitators that influence the use of low-value care [5, 16, 17]. However, this review was not able to support this recommendation since only two studies included in this review performed a barrier and facilitator assessment before executing their de-implementation strategy [9, 38]. The other studies did not describe whether they have based their de-implementation strategy on prior barrier and facilitator assessment. One study that performed a barrier assessment showed a reduction of low-value nursing care (no statistical testing) [9] and the other did not show an effective de-implementation strategy [38]. The absence of de-implementation strategies that are fully connected toward factors influencing the use of low-value nursing procedures could have contributed to ineffective de-implementation strategies in this review [17].

Another way to increase the effectiveness of de-implementation strategies may be to match de-implementation strategies to the target action (stop, replace, reduce, restrict the low-value nursing procedure) for de-implementation as different actions are underpinned by different theories, frameworks, and models for change as proposed by Norton and Chambers [17]. In this review, most studies aimed to reduce the use of restraints. Theories of habit transformation and disruption suggest that the most effective way to reduce the use of inappropriate interventions may be to change the context and environmental cues. However, studies included in this review that aimed to reduce the use of low-value restraints mostly used educational interventions (including skills training). According to theories of individual and organizational learning and unlearning strategies, this better fits with the replacement of low-value nursing procedures. Future studies should reveal whether a better match between de-implementation strategies and target actions result in more significant reductions.

This review has several strengths and limitations. The first strength is that we performed a meta-analysis to assess the effectiveness of the de-implementation strategies while Colla et al. [7] only reported whether studies were effective or not. This may have caused an overestimation of the results of the used de-implementation strategies in the review of Colla et al. [7], because the quality of the uncontrolled studies could be poor as shown in our study. Another strength is that that the number of ‘missed’ studies is limited because our search strategy was based on the 43 unique terms referring to the process of de-implementation found by Niven et al. [20] and these terms are also used in implementation studies such as ‘reduce, stop and avoid.’ Implementation studies may have the same purpose as de-implementation studies. An example of this is an implementation study that aims to implement a guideline recommendation that states ‘not to use of bandages for wounds closed by primary intention.’ In future research, the search strategy may be further improved by adding nursing procedures that are marked as low-value nursing procedures in guidelines [1, 4, 11, 12].

A limitation of this review is the quality of the included studies. The uncontrolled studies had a poor quality, which resulted in an overall low evidence based, precluding drawing conclusions. In addition, the included studies lacked measurements of patient-reported outcomes. As a result, it was not possible to determine whether the reduction of low-value nursing procedures has adverse effects on patient outcomes. Furthermore, the included studies did not report on the adherence to the intended de-implementation strategy. As a consequence, it was not possible to determine whether the de-implementation strategy has been executed as planned and the effect can be attributed to the de-implementation strategy. Therefore, further research should not only focus on developing and evaluating the effectiveness of de-implementation strategies but also to evaluate the process of the de-implementation including the identification of changes in multi-level barriers and facilitators that should be the target of the strategies [17, 53, 54]. Finally, not all controlled studies could be included in the meta-analysis due to missing data. Although we contacted the authors of the two papers with missing data on the change in volume of low-value nursing procedures, we were not able to obtain the data of two studies due to non-response of the authors.

## Conclusions

Most controlled and uncontrolled studies with a positive significant effect used a de-implementation strategy with an educational component (educational meetings, educational materials, educational outreach visits, and/or educational games) and focused their de-implementation strategy at reducing the use of restraints. Unfortunately, no conclusions can be drawn about which strategy is most effective for reducing low-value nursing.

Future studies are needed that assess whether de-implementation strategies that fully connect their strategy toward influencing factors and match their strategy to the target action (stop, replace, reduce, restrict the low value nursing procedure) are more effective for de-implementation. In order to improve future appraisal of available evidence on de-implementation strategies in nursing, we recommend that future studies should report the results on the change in the volume of low-value nursing procedures more extensively and should perform a process evaluation.

## Supplementary information

**Additional file 1.** Electronic Database Search for “Effects of de-implementation strategies aimed at reducing low-value nursing procedures: a systematic review and meta-analysis”.

## Data Availability

Abstracted data collected and analyzed during this study and described in this systematic review will be available from the corresponding author upon reasonable request.

## Abbreviations

NOS: Newcastle-Ottawa Scale
EPOC: Effective Practice and Organisation of Care
NP: Nurse practitioner
APM: Antipsychotic prescribing
LFT: Liver function test
