# Supplementary material for: Effects of de-implementation strategies aimed at reducing low-value nursing procedures: a systematic review and meta-analysis
Source: Implement Sci. 2020 May 25;15:38. doi: 10.1186/s13012-020-00995-z (PMC7249362; doi:10.1186/s13012-020-00995-z)
Supplement: Supplementary file 1 — Additional file 1. Electronic Database Search for “Effects of de-implementation strategies aimed at reducing low-value nursing procedures: a systematic review and meta-analysis”. [file 13012_2020_995_MOESM1_ESM.docx]

**Additional file 1. Electronic Database Search for “Effects of de-implementation strategies aimed at reducing low-value nursing procedures: a systematic review and meta-analysis”.**

**PUBMED**

(("Deprescriptions"[Mesh] OR Deprescription*[tw] OR De-prescription*[tw] OR Deprescri*[tw] OR De-prescri*[tw] OR (("Health Services Misuse"[Mesh] OR "overuse"[tiab] OR "overusing"[tiab] OR "overused"[tiab] OR "overuses"[tiab] OR "over use"[tiab] OR "over using"[tiab] OR "over used"[tiab] OR "over uses"[tiab] OR "over-use"[tiab] OR "over-using"[tiab] OR "over-used"[tiab] OR "over-uses"[tiab] OR "Inappropriate Prescribing"[Mesh] OR "inappropriate prescribing"[tw] OR "inappropriately prescribed"[tw] OR "inappropriate prescription"[tw] OR "choosing wisely"[tiab] OR "overtreatment"[tw] OR "overtreatments"[tw] OR "overtreating"[tw] OR "overtreated"[tw] OR "overtreats" OR "overdiagnosis"[tw] OR "overdiagnosing"[tw] OR "overdiagnoses"[tw] OR "overdiagnosed"[tw] OR "overmedication"[tw] OR "overmedicate"[tw] OR "overmedicating"[tw] OR "overmedicates"[tw] OR "overmedicated"[tw] OR (("low-value care"[ti] OR "unnecessary"[ti] OR "established"[ti] OR "ineffective"[ti] OR "practices" [ti] OR "care"[ti] OR "overuse"[ti] OR "procedure"[ti] OR "procedures"[ti]) AND ("reduction"[ti] OR "reduce"[ti] OR "reducing"[ti] OR "reduced"[ti] OR "reduces"[ti] OR "disinvestment"[ti] OR "disinvest"[ti] OR "disinvesting"[ti] OR "disinvested"[ti] OR "disinvests"[ti] OR "de-implementation"[ti] OR "de-implement"[ti] OR "de-implements"[ti] OR "de-implemented"[ti] OR "de-implementing"[ti] OR "abandoning"[ti] OR "abandon"[ti] OR "abandons"[ti] OR "abandoned"[ti] OR "abandonment"[ti] OR "discontinue"[ti] OR "disontinues"[ti] OR "discontinuing"[ti] OR "discontinued"[ti] OR "discontinuation"[ti] OR "undiffusion"[ti] OR "undiffuse"[ti] OR "undiffuses"[ti] OR "Undiffused"[ti] OR "undiffusing"[ti] OR "stop"[ti] OR "stopping"[ti] OR "stops"[ti] OR "stopped"[ti] OR "avoid"[ti] OR "avoiding"[ti] OR "avoids"[ti] OR "avoided"[ti]))))) AND ("Practice Patterns, Nurses'"[Mesh] OR "Nurses"[Mesh] OR "nurse"[tw] OR "nurses"[tw] OR "Nursing"[Mesh] OR "nursing"[tw] OR "Nurse's Role"[Mesh])) NOT (news[pt] OR comment[pt] OR editorial[pt] OR congresses[pt])

**EMBASE**

((("Deprescription"/ OR Deprescription*.mp OR De-prescription*.mp OR Deprescri*.mp OR De-prescri*.mp OR (("overuse".ti,ab OR "overusing".ti,ab OR "overused".ti,ab OR "overuses".ti,ab OR "over use".ti,ab OR "over using".ti,ab OR "over used".ti,ab OR "over uses".ti,ab OR "over-use".ti,ab OR "over-using".ti,ab OR "over-used".ti,ab OR "over-uses".ti,ab OR exp "Inappropriate Prescribing"/ OR "inappropriate prescribing".mp OR "inappropriately prescribed".mp OR "inappropriate prescription".mp OR "choosing wisely".ti,ab OR "overtreatment".mp OR "overtreatments".mp OR "overtreating".mp OR "overtreated".mp OR "overtreats".mp OR "overdiagnosis".mp OR "overdiagnosing".mp OR "overdiagnoses".mp OR "overdiagnosed".mp OR "overmedication".mp OR "overmedicate".mp OR "overmedicating".mp OR "overmedicates".mp OR "overmedicated".mp OR (("low-value care".ti OR "unnecessary".ti OR "established".ti OR "ineffective".ti OR "practices" .ti OR "care".ti OR "overuse".ti OR "procedure".ti OR "procedures".ti) AND ("reduction".ti OR "reduce".ti OR "reducing".ti OR "reduced".ti OR "reduces".ti OR "disinvestment".ti OR "disinvest".ti OR "disinvesting".ti OR "disinvested".ti OR "disinvests".ti OR "de-implementation".ti OR "de-implement".ti OR "de-implements".ti OR "de-implemented".ti OR "de-implementing".ti OR "abandoning".ti OR "abandon".ti OR "abandons".ti OR "abandoned".ti OR "abandonment".ti OR "discontinue".ti OR "disontinues".ti OR "discontinuing".ti OR "discontinued".ti OR "discontinuation".ti OR "undiffusion".ti OR "undiffuse".ti OR "undiffuses".ti OR "Undiffused".ti OR "undiffusing".ti OR "stop".ti OR "stopping".ti OR "stops".ti OR "stopped".ti OR "avoid".ti OR "avoiding".ti OR "avoids".ti OR "avoided".ti))))) AND (exp *"Nurse"/ OR "nurse".ti OR "nurses".ti OR exp *"Nursing"/ OR "nursing".ti OR *"nurse attitude"/ OR *"Nursing Practice"/)) OR ((*"Deprescription"/ OR Deprescription*.ti OR De-prescription*.ti OR Deprescri*.ti OR De-prescri*.ti OR (("overuse".ti OR "overusing".ti OR "overused".ti OR "overuses".ti OR "over use".ti OR "over using".ti OR "over used".ti OR "over uses".ti OR "over-use".ti OR "over-using".ti OR "over-used".ti OR "over-uses".ti OR exp *"Inappropriate Prescribing"/ OR "inappropriate prescribing".ti OR "inappropriately prescribed".ti OR "inappropriate prescription".ti OR "choosing wisely".ti OR "overtreatment".ti OR "overtreatments".ti OR "overtreating".ti OR "overtreated".ti OR "overtreats".ti OR "overdiagnosis".ti OR "overdiagnosing".ti OR "overdiagnoses".ti OR "overdiagnosed".ti OR "overmedication".ti OR "overmedicate".ti OR "overmedicating".ti OR "overmedicates".ti OR "overmedicated".ti OR (("low-value care".ti OR "unnecessary".ti OR "established".ti OR "ineffective".ti OR "practices" .ti OR "care".ti OR "overuse".ti OR "procedure".ti OR "procedures".ti) AND ("reduction".ti OR "reduce".ti OR "reducing".ti OR "reduced".ti OR "reduces".ti OR "disinvestment".ti OR "disinvest".ti OR "disinvesting".ti OR "disinvested".ti OR "disinvests".ti OR "de-implementation".ti OR "de-implement".ti OR "de-implements".ti OR "de-implemented".ti OR "de-implementing".ti OR "abandoning".ti OR "abandon".ti OR "abandons".ti OR "abandoned".ti OR "abandonment".ti OR "discontinue".ti OR "disontinues".ti OR "discontinuing".ti OR "discontinued".ti OR "discontinuation".ti OR "undiffusion".ti OR "undiffuse".ti OR "undiffuses".ti OR "Undiffused".ti OR "undiffusing".ti OR "stop".ti OR "stopping".ti OR "stops".ti OR "stopped".ti OR "avoid".ti OR "avoiding".ti OR "avoids".ti OR "avoided".ti))))) AND (exp "Nurse"/ OR "nurse".mp OR "nurses".mp OR exp "Nursing"/ OR "nursing".mp OR "nurse attitude"/ OR "Nursing Practice"/))) NOT (conference review or conference abstract OR editorial).pt

**Web of Science**

(TI=("Deprescriptions" OR Deprescri* OR "De prescri*" OR (("Health Services Misuse" OR "overuse" OR "overusing" OR "overused" OR "overuses" OR "over use" OR "over using" OR "over used" OR "over uses" OR "over-use" OR "over-using" OR "over-used" OR "over-uses" OR "inappropriate prescribing" OR "inappropriately prescribed" OR "inappropriate prescription" OR "choosing wisely" OR "overtreatment" OR "overtreatments" OR "overtreating" OR "overtreated" OR "overtreats" OR "overdiagnosis" OR "overdiagnosing" OR "overdiagnoses" OR "overdiagnosed" OR "overmedication" OR "overmedicate" OR "overmedicating" OR "overmedicates" OR "overmedicated" OR (("low-value care" OR "unnecessary" OR "established" OR "ineffective" OR "practices" OR "care" OR "overuse" OR "procedure" OR "procedures") AND ("reduction" OR "reduce" OR "reducing" OR "reduced" OR "reduces" OR "disinvestment" OR "disinvest" OR "disinvesting" OR "disinvested" OR "disinvests" OR "de-implementation" OR "de-implement" OR "de-implements" OR "de-implemented" OR "de-implementing" OR "abandoning" OR "abandon" OR "abandons" OR "abandoned" OR "abandonment" OR "discontinue" OR "disontinues" OR "discontinuing" OR "discontinued" OR "discontinuation" OR "undiffusion" OR "undiffuse" OR "undiffuses" OR "Undiffused" OR "undiffusing" OR "stop" OR "stopping" OR "stops" OR "stopped" OR "avoid" OR "avoiding" OR "avoids" OR "avoided"))))) AND TS=("Nurses" OR "nurse" OR "nursing"))

**Cochrane**

(("Deprescriptions" OR Deprescri* OR "De prescri*" OR (("Health Services Misuse" OR "overuse" OR "overusing" OR "overused" OR "overuses" OR "over use" OR "over using" OR "over used" OR "over uses" OR "over-use" OR "over-using" OR "over-used" OR "over-uses" OR "inappropriate prescribing" OR "inappropriately prescribed" OR "inappropriate prescription" OR "choosing wisely" OR "overtreatment" OR "overtreatments" OR "overtreating" OR "overtreated" OR "overtreats" OR "overdiagnosis" OR "overdiagnosing" OR "overdiagnoses" OR "overdiagnosed" OR "overmedication" OR "overmedicate" OR "overmedicating" OR "overmedicates" OR "overmedicated" OR (("low-value care" OR "unnecessary" OR "established" OR "ineffective" OR "practices" OR "care" OR "overuse" OR "procedure" OR "procedures") AND ("reduction" OR "reduce" OR "reducing" OR "reduced" OR "reduces" OR "disinvestment" OR "disinvest" OR "disinvesting" OR "disinvested" OR "disinvests" OR "de-implementation" OR "de-implement" OR "de-implements" OR "de-implemented" OR "de-implementing" OR "abandoning" OR "abandon" OR "abandons" OR "abandoned" OR "abandonment" OR "discontinue" OR "disontinues" OR "discontinuing" OR "discontinued" OR "discontinuation" OR "undiffusion" OR "undiffuse" OR "undiffuses" OR "Undiffused" OR "undiffusing" OR "stop" OR "stopping" OR "stops" OR "stopped" OR "avoid" OR "avoiding" OR "avoids" OR "avoided"))))):ti AND ("Nurses" OR "nurse" OR "nursing"):ti,ab,kw)

**Emcare**

(("Deprescription"/ OR Deprescription*.mp OR De-prescription*.mp OR Deprescri*.mp OR De-prescri*.mp OR (("overuse".ti,ab. OR "overusing".ti,ab. OR "overused".ti,ab. OR "overuses".ti,ab. OR "over use".ti,ab. OR "over using".ti,ab. OR "over used".ti,ab. OR "over uses".ti,ab. OR "over-use".ti,ab. OR "over-using".ti,ab. OR "over-used".ti,ab. OR "over-uses".ti,ab. OR exp "Inappropriate Prescribing"/ OR "inappropriate prescribing".mp OR "inappropriately prescribed".mp OR "inappropriate prescription".mp OR "choosing wisely".ti,ab. OR "overtreatment".mp OR "overtreatments".mp OR "overtreating".mp OR "overtreated".mp OR "overtreats".mp OR "overdiagnosis".mp OR "overdiagnosing".mp OR "overdiagnoses".mp OR "overdiagnosed".mp OR "overmedication".mp OR "overmedicate".mp OR "overmedicating".mp OR "overmedicates".mp OR "overmedicated".mp OR (("low-value care".ti OR "unnecessary".ti OR "established".ti OR "ineffective".ti OR "practices" .ti OR "care".ti OR "overuse".ti OR "procedure".ti OR "procedures".ti) AND ("reduction".ti OR "reduce".ti OR "reducing".ti OR "reduced".ti OR "reduces".ti OR "disinvestment".ti OR "disinvest".ti OR "disinvesting".ti OR "disinvested".ti OR "disinvests".ti OR "de-implementation".ti OR "de-implement".ti OR "de-implements".ti OR "de-implemented".ti OR "de-implementing".ti OR "abandoning".ti OR "abandon".ti OR "abandons".ti OR "abandoned".ti OR "abandonment".ti OR "discontinue".ti OR "disontinues".ti OR "discontinuing".ti OR "discontinued".ti OR "discontinuation".ti OR "undiffusion".ti OR "undiffuse".ti OR "undiffuses".ti OR "Undiffused".ti OR "undiffusing".ti OR "stop".ti OR "stopping".ti OR "stops".ti OR "stopped".ti OR "avoid".ti OR "avoiding".ti OR "avoids".ti OR "avoided".ti))))) AND (exp "Nurse"/ OR "nurse".mp OR "nurses".mp OR exp "Nursing"/ OR "nursing".mp OR "nurse attitude"/ OR "Nursing Practice"/)) NOT (conference review or conference abstract).pt

**PsycINFO**

TI(("Deprescriptions" OR "depresciption" OR depresci* OR (( "overuse" OR "overuse" OR "overusing" OR "overusing" OR "overused" OR "overused" OR "overuses" OR "overuses" OR "over use" OR "over use" OR "over using" OR "over using" OR "over used" OR "over used" OR "over uses" OR "over uses"OR "over-use" OR "over-use" OR "over-using" OR "over-using" OR "over-used" OR "over-used" OR "over-uses" OR "over-uses" OR "inappropriate prescribing" OR "inappropriately prescribed" OR "inappropriate prescription" OR "choosing wisely" OR "choosing wisely" OR "overtreatment" OR "overtreatments" OR "overtreating" OR "overtreated" OR "overtreats" OR "overdiagnosis" OR "overdiagnosing" OR "overdiagnoses" OR "overdiagnosed" OR "overmedication" OR "overmedicate" OR "overmedicating" OR "overmedicates" OR "overmedicated" OR (( "low-value care" OR "unnecessary" OR "established" OR "ineffective" OR "practices" OR "care" OR "overuse" OR "procedure" OR "procedures") AND ( "reduction" OR "reduce" OR "reducing" OR "reduced" OR "reduces" OR "disinvestment" OR "disinvest" OR "disinvesting" OR "disinvested" OR "disinvests" OR "de-implementation" OR "de-implement" OR "de-implements" OR "de-implemented" OR "de-implementing" OR "abandoning" OR "abandon" OR "abandons" OR "abandoned" OR "abandonment" OR "discontinue" OR "disontinues" OR "discontinuing" OR "discontinued" OR "discontinuation" OR "undiffusion" OR "undiffuse" OR "undiffuses" OR "Undiffused" OR "undiffusing" OR "stop" OR "stopping" OR "stops" OR "stopped" OR "avoid" OR "avoiding" OR "avoids" OR "avoided"))))) AND ("Nurses" OR "Psychiatric Nurses" OR "Public Health Service Nurses" OR "School Nurses" OR "nurse" OR "nurses" OR "Nursing" OR "nursing")) OR (TI("Deprescriptions" OR "depresciption" OR depresci* OR (( "overuse" OR "overuse" OR "overusing" OR "overusing" OR "overused" OR "overused" OR "overuses" OR "overuses" OR "over use" OR "over use" OR "over using" OR "over using" OR "over used" OR "over used" OR "over uses" OR "over uses"OR "over-use" OR "over-use" OR "over-using" OR "over-using" OR "over-used" OR "over-used" OR "over-uses" OR "over-uses" OR "inappropriate prescribing" OR "inappropriately prescribed" OR "inappropriate prescription" OR "choosing wisely" OR "choosing wisely" OR "overtreatment" OR "overtreatments" OR "overtreating" OR "overtreated" OR "overtreats" OR "overdiagnosis" OR "overdiagnosing" OR "overdiagnoses" OR "overdiagnosed" OR "overmedication" OR "overmedicate" OR "overmedicating" OR "overmedicates" OR "overmedicated" OR (( "low-value care" OR "unnecessary" OR "established" OR "ineffective" OR "practices" OR "care" OR "overuse" OR "procedure" OR "procedures") AND ( "reduction" OR "reduce" OR "reducing" OR "reduced" OR "reduces" OR "disinvestment" OR "disinvest" OR "disinvesting" OR "disinvested" OR "disinvests" OR "de-implementation" OR "de-implement" OR "de-implements" OR "de-implemented" OR "de-implementing" OR "abandoning" OR "abandon" OR "abandons" OR "abandoned" OR "abandonment" OR "discontinue" OR "disontinues" OR "discontinuing" OR "discontinued" OR "discontinuation" OR "undiffusion" OR "undiffuse" OR "undiffuses" OR "Undiffused" OR "undiffusing" OR "stop" OR "stopping" OR "stops" OR "stopped" OR "avoid" OR "avoiding" OR "avoids" OR "avoided"))))) AND DE("Nurses" OR "Psychiatric Nurses" OR "Public Health Service Nurses" OR "School Nurses" OR "nurse" OR "nurses" OR "Nursing" OR "nursing")) OR (TI("Deprescriptions" OR "depresciption" OR depresci* OR (( "overuse" OR "overuse" OR "overusing" OR "overusing" OR "overused" OR "overused" OR "overuses" OR "overuses" OR "over use" OR "over use" OR "over using" OR "over using" OR "over used" OR "over used" OR "over uses" OR "over uses"OR "over-use" OR "over-use" OR "over-using" OR "over-using" OR "over-used" OR "over-used" OR "over-uses" OR "over-uses" OR "inappropriate prescribing" OR "inappropriately prescribed" OR "inappropriate prescription" OR "choosing wisely" OR "choosing wisely" OR "overtreatment" OR "overtreatments" OR "overtreating" OR "overtreated" OR "overtreats" OR "overdiagnosis" OR "overdiagnosing" OR "overdiagnoses" OR "overdiagnosed" OR "overmedication" OR "overmedicate" OR "overmedicating" OR "overmedicates" OR "overmedicated" OR (( "low-value care" OR "unnecessary" OR "established" OR "ineffective" OR "practices" OR "care" OR "overuse" OR "procedure" OR "procedures") AND ( "reduction" OR "reduce" OR "reducing" OR "reduced" OR "reduces" OR "disinvestment" OR "disinvest" OR "disinvesting" OR "disinvested" OR "disinvests" OR "de-implementation" OR "de-implement" OR "de-implements" OR "de-implemented" OR "de-implementing" OR "abandoning" OR "abandon" OR "abandons" OR "abandoned" OR "abandonment" OR "discontinue" OR "disontinues" OR "discontinuing" OR "discontinued" OR "discontinuation" OR "undiffusion" OR "undiffuse" OR "undiffuses" OR "Undiffused" OR "undiffusing" OR "stop" OR "stopping" OR "stops" OR "stopped" OR "avoid" OR "avoiding" OR "avoids" OR "avoided"))))) AND AB("Nurses" OR "Psychiatric Nurses" OR "Public Health Service Nurses" OR "School Nurses" OR "nurse" OR "nurses" OR "Nursing" OR "nursing"))

**Academic Search Premier**

TI(("Deprescriptions" OR "depresciption" OR depresci* OR (( "overuse" OR "overuse" OR "overusing" OR "overusing" OR "overused" OR "overused" OR "overuses" OR "overuses" OR "over use" OR "over use" OR "over using" OR "over using" OR "over used" OR "over used" OR "over uses" OR "over uses"OR "over-use" OR "over-use" OR "over-using" OR "over-using" OR "over-used" OR "over-used" OR "over-uses" OR "over-uses" OR "inappropriate prescribing" OR "inappropriately prescribed" OR "inappropriate prescription" OR "choosing wisely" OR "choosing wisely" OR "overtreatment" OR "overtreatments" OR "overtreating" OR "overtreated" OR "overtreats" OR "overdiagnosis" OR "overdiagnosing" OR "overdiagnoses" OR "overdiagnosed" OR "overmedication" OR "overmedicate" OR "overmedicating" OR "overmedicates" OR "overmedicated" OR (( "low-value care" OR "unnecessary" OR "established" OR "ineffective" OR "practices" OR "care" OR "overuse" OR "procedure" OR "procedures") AND ( "reduction" OR "reduce" OR "reducing" OR "reduced" OR "reduces" OR "disinvestment" OR "disinvest" OR "disinvesting" OR "disinvested" OR "disinvests" OR "de-implementation" OR "de-implement" OR "de-implements" OR "de-implemented" OR "de-implementing" OR "abandoning" OR "abandon" OR "abandons" OR "abandoned" OR "abandonment" OR "discontinue" OR "disontinues" OR "discontinuing" OR "discontinued" OR "discontinuation" OR "undiffusion" OR "undiffuse" OR "undiffuses" OR "Undiffused" OR "undiffusing" OR "stop" OR "stopping" OR "stops" OR "stopped" OR "avoid" OR "avoiding" OR "avoids" OR "avoided"))))) AND ("Nurses" OR "Psychiatric Nurses" OR "Public Health Service Nurses" OR "School Nurses" OR "nurse" OR "nurses" OR "Nursing" OR "nursing")) OR (TI("Deprescriptions" OR "depresciption" OR depresci* OR (( "overuse" OR "overuse" OR "overusing" OR "overusing" OR "overused" OR "overused" OR "overuses" OR "overuses" OR "over use" OR "over use" OR "over using" OR "over using" OR "over used" OR "over used" OR "over uses" OR "over uses"OR "over-use" OR "over-use" OR "over-using" OR "over-using" OR "over-used" OR "over-used" OR "over-uses" OR "over-uses" OR "inappropriate prescribing" OR "inappropriately prescribed" OR "inappropriate prescription" OR "choosing wisely" OR "choosing wisely" OR "overtreatment" OR "overtreatments" OR "overtreating" OR "overtreated" OR "overtreats" OR "overdiagnosis" OR "overdiagnosing" OR "overdiagnoses" OR "overdiagnosed" OR "overmedication" OR "overmedicate" OR "overmedicating" OR "overmedicates" OR "overmedicated" OR (( "low-value care" OR "unnecessary" OR "established" OR "ineffective" OR "practices" OR "care" OR "overuse" OR "procedure" OR "procedures") AND ( "reduction" OR "reduce" OR "reducing" OR "reduced" OR "reduces" OR "disinvestment" OR "disinvest" OR "disinvesting" OR "disinvested" OR "disinvests" OR "de-implementation" OR "de-implement" OR "de-implements" OR "de-implemented" OR "de-implementing" OR "abandoning" OR "abandon" OR "abandons" OR "abandoned" OR "abandonment" OR "discontinue" OR "disontinues" OR "discontinuing" OR "discontinued" OR "discontinuation" OR "undiffusion" OR "undiffuse" OR "undiffuses" OR "Undiffused" OR "undiffusing" OR "stop" OR "stopping" OR "stops" OR "stopped" OR "avoid" OR "avoiding" OR "avoids" OR "avoided"))))) AND KW("Nurses" OR "Psychiatric Nurses" OR "Public Health Service Nurses" OR "School Nurses" OR "nurse" OR "nurses" OR "Nursing" OR "nursing")) OR (TI("Deprescriptions" OR "depresciption" OR depresci* OR (( "overuse" OR "overuse" OR "overusing" OR "overusing" OR "overused" OR "overused" OR "overuses" OR "overuses" OR "over use" OR "over use" OR "over using" OR "over using" OR "over used" OR "over used" OR "over uses" OR "over uses"OR "over-use" OR "over-use" OR "over-using" OR "over-using" OR "over-used" OR "over-used" OR "over-uses" OR "over-uses" OR "inappropriate prescribing" OR "inappropriately prescribed" OR "inappropriate prescription" OR "choosing wisely" OR "choosing wisely" OR "overtreatment" OR "overtreatments" OR "overtreating" OR "overtreated" OR "overtreats" OR "overdiagnosis" OR "overdiagnosing" OR "overdiagnoses" OR "overdiagnosed" OR "overmedication" OR "overmedicate" OR "overmedicating" OR "overmedicates" OR "overmedicated" OR (( "low-value care" OR "unnecessary" OR "established" OR "ineffective" OR "practices" OR "care" OR "overuse" OR "procedure" OR "procedures") AND ( "reduction" OR "reduce" OR "reducing" OR "reduced" OR "reduces" OR "disinvestment" OR "disinvest" OR "disinvesting" OR "disinvested" OR "disinvests" OR "de-implementation" OR "de-implement" OR "de-implements" OR "de-implemented" OR "de-implementing" OR "abandoning" OR "abandon" OR "abandons" OR "abandoned" OR "abandonment" OR "discontinue" OR "disontinues" OR "discontinuing" OR "discontinued" OR "discontinuation" OR "undiffusion" OR "undiffuse" OR "undiffuses" OR "Undiffused" OR "undiffusing" OR "stop" OR "stopping" OR "stops" OR "stopped" OR "avoid" OR "avoiding" OR "avoids" OR "avoided"))))) AND AB("Nurses" OR "Psychiatric Nurses" OR "Public Health Service Nurses" OR "School Nurses" OR "nurse" OR "nurses" OR "Nursing" OR "nursing"))

**CINAHL**

TI(("Deprescriptions" OR "depresciption" OR depresci* OR (( "overuse" OR "overuse" OR "overusing" OR "overusing" OR "overused" OR "overused" OR "overuses" OR "overuses" OR "over use" OR "over use" OR "over using" OR "over using" OR "over used" OR "over used" OR "over uses" OR "over uses"OR "over-use" OR "over-use" OR "over-using" OR "over-using" OR "over-used" OR "over-used" OR "over-uses" OR "over-uses" OR "inappropriate prescribing" OR "inappropriately prescribed" OR "inappropriate prescription" OR "choosing wisely" OR "choosing wisely" OR "overtreatment" OR "overtreatments" OR "overtreating" OR "overtreated" OR "overtreats" OR "overdiagnosis" OR "overdiagnosing" OR "overdiagnoses" OR "overdiagnosed" OR "overmedication" OR "overmedicate" OR "overmedicating" OR "overmedicates" OR "overmedicated" OR (("low-value care" OR "unnecessary" OR "established" OR "ineffective" OR "practices" OR "care" OR "overuse" OR "procedure" OR "procedures") AND ("reduction" OR "reduce" OR "reducing" OR "reduced" OR "reduces" OR "disinvestment" OR "disinvest" OR "disinvesting" OR "disinvested" OR "disinvests" OR "de-implementation" OR "de-implement" OR "de-implements" OR "de-implemented" OR "de-implementing" OR "abandoning" OR "abandon" OR "abandons" OR "abandoned" OR "abandonment" OR "discontinue" OR "disontinues" OR "discontinuing" OR "discontinued" OR "discontinuation" OR "undiffusion" OR "undiffuse" OR "undiffuses" OR "Undiffused" OR "undiffusing" OR "stop" OR "stopping" OR "stops" OR "stopped" OR "avoid" OR "avoiding" OR "avoids" OR "avoided"))))) AND ("Nurses" OR "Psychiatric Nurses" OR "Public Health Service Nurses" OR "School Nurses" OR "nurse" OR "nurses" OR "Nursing" OR "nursing")) OR (TI("Deprescriptions" OR "depresciption" OR depresci* OR (( "overuse" OR "overuse" OR "overusing" OR "overusing" OR "overused" OR "overused" OR "overuses" OR "overuses" OR "over use" OR "over use" OR "over using" OR "over using" OR "over used" OR "over used" OR "over uses" OR "over uses"OR "over-use" OR "over-use" OR "over-using" OR "over-using" OR "over-used" OR "over-used" OR "over-uses" OR "over-uses" OR "inappropriate prescribing" OR "inappropriately prescribed" OR "inappropriate prescription" OR "choosing wisely" OR "choosing wisely" OR "overtreatment" OR "overtreatments" OR "overtreating" OR "overtreated" OR "overtreats" OR "overdiagnosis" OR "overdiagnosing" OR "overdiagnoses" OR "overdiagnosed" OR "overmedication" OR "overmedicate" OR "overmedicating" OR "overmedicates" OR "overmedicated" OR (("low-value care" OR "unnecessary" OR "established" OR "ineffective" OR "practices" OR "care" OR "overuse" OR "procedure" OR "procedures") AND ("reduction" OR "reduce" OR "reducing" OR "reduced" OR "reduces" OR "disinvestment" OR "disinvest" OR "disinvesting" OR "disinvested" OR "disinvests" OR "de-implementation" OR "de-implement" OR "de-implements" OR "de-implemented" OR "de-implementing" OR "abandoning" OR "abandon" OR "abandons" OR "abandoned" OR "abandonment" OR "discontinue" OR "disontinues" OR "discontinuing" OR "discontinued" OR "discontinuation" OR "undiffusion" OR "undiffuse" OR "undiffuses" OR "Undiffused" OR "undiffusing" OR "stop" OR "stopping" OR "stops" OR "stopped" OR "avoid" OR "avoiding" OR "avoids" OR "avoided"))))) AND DE("Nurses" OR "Psychiatric Nurses" OR "Public Health Service Nurses" OR "School Nurses" OR "nurse" OR "nurses" OR "Nursing" OR "nursing")) OR (TI("Deprescriptions" OR "depresciption" OR depresci* OR (( "overuse" OR "overuse" OR "overusing" OR "overusing" OR "overused" OR "overused" OR "overuses" OR "overuses" OR "over use" OR "over use" OR "over using" OR "over using" OR "over used" OR "over used" OR "over uses" OR "over uses"OR "over-use" OR "over-use" OR "over-using" OR "over-using" OR "over-used" OR "over-used" OR "over-uses" OR "over-uses" OR "inappropriate prescribing" OR "inappropriately prescribed" OR "inappropriate prescription" OR "choosing wisely" OR "choosing wisely" OR "overtreatment" OR "overtreatments" OR "overtreating" OR "overtreated" OR "overtreats" OR "overdiagnosis" OR "overdiagnosing" OR "overdiagnoses" OR "overdiagnosed" OR "overmedication" OR "overmedicate" OR "overmedicating" OR "overmedicates" OR "overmedicated" OR (("low-value care" OR "unnecessary" OR "established" OR "ineffective" OR "practices" OR "care" OR "overuse" OR "procedure" OR "procedures") AND ( "reduction" OR "reduce" OR "reducing" OR "reduced" OR "reduces" OR "disinvestment" OR "disinvest" OR "disinvesting" OR "disinvested" OR "disinvests" OR "de-implementation" OR "de-implement" OR "de-implements" OR "de-implemented" OR "de-implementing" OR "abandoning" OR "abandon" OR "abandons" OR "abandoned" OR "abandonment" OR "discontinue" OR "disontinues" OR "discontinuing" OR "discontinued" OR "discontinuation" OR "undiffusion" OR "undiffuse" OR "undiffuses" OR "Undiffused" OR "undiffusing" OR "stop" OR "stopping" OR "stops" OR "stopped" OR "avoid" OR "avoiding" OR "avoids" OR "avoided"))))) AND AB("Nurses" OR "Psychiatric Nurses" OR "Public Health Service Nurses" OR "School Nurses" OR "nurse" OR "nurses" OR "Nursing" OR "nursing"))

**Google Scholar**

Deprescriptions|"Health Services Misuse"|"overuse"|"inappropriate prescribing" "choosing wisely"|"overtreatment"|"overdiagnosis"|"overmedication" "Nurse"|"nurse"|"nursing"

"low-value care"|"unnecessary"|"established"|"ineffective"|"overuse"|"procedure"|"procedures" "de-implementation"|"de-implement"|"de-implements"|"de-implemented"|"de-implementing"|"abandon"|"discontinue" "Nurse"|"nurse"|"nursing
